# Therapeutic implications of current Janus kinase inhibitors as anti-COVID agents: A review

**DOI:** 10.3389/fphar.2023.1135145

**Published:** 2023-03-20

**Authors:** Nem Kumar Jain, Mukul Tailang, Hemant Kumar Jain, Balakumar Chandrasekaran, Biswa Mohan Sahoo, Anandhalakshmi Subramanian, Neelaveni Thangavel, Afaf Aldahish, Kumarappan Chidambaram, M. Alagusundaram, Santosh Kumar, Palani Selvam

**Affiliations:** ^1^ School of Pharmacy, ITM University, Gwalior, Madhya Pradesh, India; ^2^ School of Studies in Pharmaceutical Sciences, Jiwaji University, Gwalior, Madhya Pradesh, India; ^3^ Department of General Medicine, Government Medical College, Datia, Madhya Pradesh, India; ^4^ Faculty of Pharmacy, Philadelphia University, Amman, Jordan; ^5^ Roland Institute of Pharmaceutical Sciences, Berhampur, Odisha, India; ^6^ Department of Microbiology and Clinical Parasitology, College of Medicine, King Khalid University, Abha, Saudi Arabia; ^7^ Department of Pharmaceutical Chemistry and Pharmacognosy, College of Pharmacy, Jazan University, Jazan, Saudi Arabia; ^8^ Department of Pharmacology, College of Pharmacy, King Khalid University, Abha, Saudi Arabia; ^9^ School of Sciences, ITM University, Gwalior, Madhya Pradesh, India; ^10^ School of Medicine, College of Medicine and Health Sciences, Jijiga University, Jijiga, Ethiopia

**Keywords:** Janus kinase, JAKs, JAK-STAT pathway, JAKi, Jakinibs, kinase inhibitors, COVID-19, SARS-CoV-2

## Abstract

Severe cases of COVID-19 are characterized by hyperinflammation induced by cytokine storm, ARDS leading to multiorgan failure and death. JAK-STAT signaling has been implicated in immunopathogenesis of COVID-19 infection under different stages such as viral entry, escaping innate immunity, replication, and subsequent inflammatory processes. Prompted by this fact and prior utilization as an immunomodulatory agent for several autoimmune, allergic, and inflammatory conditions, Jakinibs have been recognized as validated small molecules targeting the rapid release of proinflammatory cytokines, primarily IL-6, and GM-CSF. Various clinical trials are under investigation to evaluate Jakinibs as potential candidates for treating COVID-19. Till date, there is only one small molecule Jakinib known as baricitinib has received FDA-approval as a standalone immunomodulatory agent in treating critical COVID-19 patients. Though various meta-analyses have confirmed and validated the safety and efficacy of Jakinibs, further studies are required to understand the elaborated pathogenesis of COVID-19, duration of Jakinib treatment, and assess the combination therapeutic strategies. In this review, we highlighted JAK-STAT signalling in the pathogenesis of COVID-19 and clinically approved Jakinibs. Moreover, this review described substantially the promising use of Jakinibs and discussed their limitations in the context of COVID-19 therapy. Hence, this review article provides a concise, yet significant insight into the therapeutic implications of Jakinibs as potential anti-COVID agents which opens up a new horizon in the treatment of COVID-19, effectively.

## 1 Introduction

Over the last 30 years, research has established Janus kinases (JAKs) as a lucrative target for drug development against various autoimmune, allergic, and inflammatory diseases. Wilks, (1989) started this journey of JAKs when he reported for the time JAKs as a new class of protein-tyrosine kinase (PTK) family, discovered using PCR-based strategies or low stringency hybridization techniques. Initially, JAKs was named “Just another kinase.” Later, based on the tandem architecture of kinase domains renamed Janus Kinase in reference to the two-faced Roman god Janus (Fragoulis et al., 2019).

JAKs are part of the intracellular non-receptor PTK family, acting as signal transducers within the cell and include four mammalian members (JAK1, JAK2, JAK3, and TYK2) (Garcia-Melendo et al., 2021). JAK1-2, and TYK2 are ubiquitously expressed in mammalian cells, while JAK3 expression is limited to the hematopoietic and lymphatic systems. JAK1 and JAK2 genes in humans are located on chromosomes 1p31.3 and 9p24, respectively, while the JAK3 and TYK2 genes are located on chromosomes 19p13.1 and 19p13.2, respectively. JAKs are also found in birds, fish, mice, and invertebrates. (Yamaoka et al., 2004).

These four tyrosine kinase proteins are essential in transducing the almost 57 types of cytokine-mediated signaling *via* Janus kinase (JAK)-signal transducers and activators of the transcription (STAT) pathway. Due to their role in cytokine-mediated immunopathologic changes, the JAK-STAT pathway has emerged as a potential drug target in many autoimmune, allergic, and inflammatory conditions, including cancers. The Janus Kinase inhibitors (Jakinibs) are one of the three therapeutic modalities to target the JAK-STAT pathway in cytokine signaling; other modalities are cytokine or receptor antagonists and STAT inhibitors (Mortezavi et al., 2022). The past decade has witnessed the remarkable success of Jakinibs. Since the first approval of the first Jakinib that is ruxolitinib, for myeloproliferative disorders, a total of 12 Jakinibs have been approved for an array of autoimmune and inflammatory conditions.

Coronavirus-2019 (COVID-19) pandemic is caused by severe acute respiratory syndrome-coronavirus-2 (SARS-CoV-2) and is characterized by a respiratory illness, multiple organ failure, pneumonia, acute respiratory distress syndrome (ARDS), and finally, death (Agrawal et al., 2020; Jain et al., 2022). In addition to these symptoms, patients with this condition exhibit several systemic manifestations, such as lymphopenia, abnormalities of ferritin levels, prothrombin time, platelet count, acute partial thromboplastin time, and blood clotting anomalies (Zhang et al., 2023). SARS-CoV-2-induced senescence of infected cells was reported to be a triggering factor for COVID-19-related cytokine release syndrome and multi-organ failure (Lee et al., 2021).

Based on disease severity and clinical manifestations, drugs may be selected for COVID-19 treatment from broad therapeutic categories, including antiviral agents, immunosuppressive agents, anticoagulants, and other adjuvant therapies. Antiviral agents are recommended in the early stage of viral infection to prevent proliferation and eliminate viruses. These agents are primarily indicated for patients with mild-to-moderate disease and have limited effectiveness in advanced pneumonia, ARDS, and severe inflammation caused by a cytokine storm (van de Veerdonk et al., 2022). The only antiviral medication recommended thus far for COVID-19 therapy is Molnupiravir (Mahase, 2021). Due to the continuous triggering of innate and acquired immune systems and elevated production of pro-inflammatory mediators associated with severe and critical cases of COVID-19, various immunomodulatory and immunosuppressive agents such as monoclonal antibodies and interleukin antagonists have been investigated in clinical trials (Gantla et al., 2023). Antibodies targeting IL-6 or GM-CSF, such as tocilizumab, sarilumab, gimsilumab, and lenzilumab, are approved or under clinical investigations to treat severe COVID-19 patients (Agrawal et al., 2021; D’Alessio et al., 2021). However, these drugs usually target one cytokine, and their benefits are equivocal in the clinical setting. Severe and critical COVID-19 cases also demand respiratory therapies such as oxygen therapy, mechanical ventilator support, and extracorporeal membrane oxygenation (ECMO) as adjuvant therapy (Frat et al., 2023). Glucocorticoids as immunosuppressive agents have been used for respiratory pneumonia due to COVID-19; however, the use is controversial due to inherent immunosuppression-associated adverse reactions and increased risk of infection, disease worsening, and poor prognosis (Tam et al., 2021). The treatment armamentarium of COVID-19 is rapidly evolving. Therapies such as convalescent plasma, corticosteroids, IL-6 antagonists, and other anti-SARS-CoV-2 monoclonal antibodies are either approved or recommended to improve prognosis and reduce the clinical burden of COVID-19. Jakinibs are the recent addition to the treatment landscape of COVID-19; while their role is promising but remains to be established (Al-Hajeri et al., 2022).

This review aimed to describe the progress in understanding JAK-STAT pathway signaling in the pathogenesis of less obvious setting COVID-19. Due to their dual anti-viral and anti-inflammatory potential, various reports have indicated the suitability of Jakinibs to be repurposed for COVID-19 infection. This review will further recapitulate the current clinical development status regarding applying Jakinibs in COVID-19. An update on global approvals of various Jakinibs against multiple autoimmune and inflammatory disorders is also appended.

### 1.1 Janus kinases (JAKs)

**FIGURE 1:**
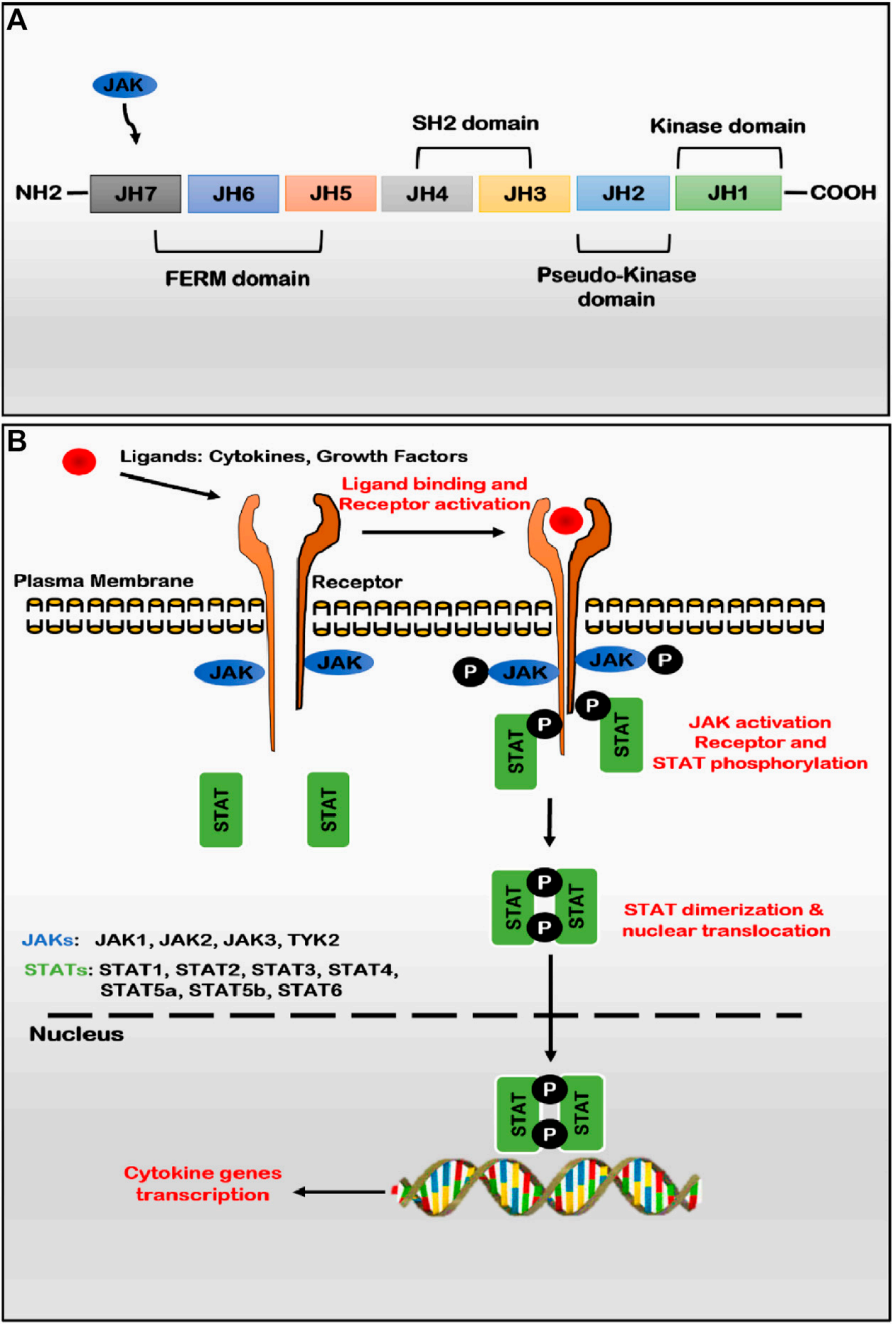
**(A)** JAK structure: seven homology domains (JH) constitute the structure of four structural domains; JH1 is an active catalytic domain (kinase) regulated by the JH2 domain (pseudo-kinase), FERM and SH2 domains involved with maintaining the kinase structure and binding to cytokine receptors **(B)** JAK-STAT pathway: binding of several ligands to membrane-bound cytokine or hormone receptors leading to receptor dimerization and activation of constitutively associated or recruited JAKs *via* auto and/or trans-phosphorylation of tyrosine residues on the activation loop of the kinase domain. Activated JAKs function as dimers (homo- or heterodimers) and phosphorylates receptor chains that serve as the docking site for cytoplasmic and recruited STAT factors. Once phosphorylated, STAT factors get dimerized (homo- or heterodimers) and dissociate from receptors to translocate and accumulate in the nucleus to activate or suppress the transcription of various cytokine genes. (Abbreviations: JAK, Janus kinase; STAT, signal transducer and activator of transcription).

JAKs are relatively large proteins that range between 120–140 kDa in molecular mass, consisting of more than 1,100 amino acids. Structurally, the primary structure of JAKs exhibits seven evolutionary conserved Janus homology (JH) domains (JH1-JH7), which putatively fold into four domains viz. FERM (band four points one, ezrin, radixin, and moesin), Src Homology-2 like (SH2-like), pseudo-kinase and kinase domains (Figure 1A) (Behrmann et al., 2004). At the C-terminal of the enzyme, JH1 and JH2 regions encode active catalytic kinase and pseud-kinase domains, respectively. JH1 region is responsible for the enzymatic activity of JAKs, while the JH2 domain (pseudo-kinase) is characterized by dual kinase specificity and negatively regulates active catalytic tyrosine kinase activity (JH1) (Lupardus et al., 2014). Other domains like FERM (JH5-JH7) and SH2-like (JH3-JH4) do not exhibit enzymatic activity but are predicted to be essential for interacting with three regions, namely, box 1, interbox, and box 2 of cytokine receptor tails (Ghoreschi et al., 2009b). The JAKs-FERM is located at the amino terminus of JAKs, is a 300 amino acids long domain which resembles canonical FERM and consists of three sub-domains such as ubiquitin-like F1, acyl CoA-binding proteins like F2 and pleckstrin homology domain like F1, F2, and F3 jointly form a compact clover shape structure (Wallweber et al., 2014). Like pseudo-kinase, FERM binds to the tyrosine kinase domain and positively regulates catalytic activity by maintaining an active state. The three-dimensional structure of JAKs is still unclear; however, the crystal structure of the JH1 (kinase) domain for all JAKs has been determined in the active state conformation, depicting bilobed fold characteristics. JAKs exhibit autophosphorylation at multiple sites, including one each in the FERM and pseudo-kinase domain and two sites in the catalytic active kinase domain. No binding partners have been identified for the SH2 domain (Yamaoka et al., 2004). JAKs convey signals from various ligand-receptor complexes, such as cytokines, interferons, and growth hormones, to the nucleus, producing many bioactive compounds that alter cell metabolism and functions. Due to such ability, JAKs play an essential role in adequately functioning innate and adaptive immunity and proper conduction of physiological processes such as haematopoiesis, maturation of immune cells, and many others (O’Shea and Plenge, 2012; Seif et al., 2017a).

A variety of cytokine receptors (both Class I and Class II) utilize JAKs as the preferred transducer mechanism to promote the transcription of ligand-related genes in multiple pathogeneses (Schwartz et al., 2016). Other receptors associated with JAKs activation include growth factor receptors, FGFR, PDGFR, VEGFR, and hormone receptors for growth hormone, prolactin, thrombopoietin and erythropoietin (Raju et al., 2014). JAK1 and JAK3 have been reported to be crucial for lymphopoiesis, while JAK2 and TYK2 are crucial for IL-12 dependent T-cell differentiation. Type I cytokine receptors with typical *γ* chain like IL-2R, IL-4R, IL-7R, IL-9R, IL-15R, and IL-21R activates JAK1 and JAK3 to trigger the JAK/STAT pathway (Notarangelo et al., 2001; O’Shea et al., 2004; Spinelli et al., 2021). JAK3 is exclusively linked to immune cells like B-cells, T-cells, NK-cells, and monocytes (Cornejo et al., 2009; Uckun et al., 2011). Stimulation of cytokine receptors including IL-10R, IL-19R, IL-20R, IL-22R, IL-6R, IL-11R, and GMCSFR results in activation of JAK1 and JAK2 kinases. JAK-2 is exclusive to signal transmission for cytokine receptors, including IL-3R, hormone receptors for growth hormone, prolactin, erythropoietin, etc., (Sopjani et al., 2021). TYK3 forms heterodimers with JAK1 or JAK2 kinases for signal transduction. Type I interferon receptors use JAK2 and TYK2, while p40-containing cytokine receptors, such as IL-12R and IL-23R, use JAK2 and TYK2 kinases together (Gonciarz et al., 2021). Various other receptors, such as receptor tyrosine kinases, death receptors (CD40), and G-protein coupled receptors (chemokine receptors), have also been implicated in JAKs kinase activation (Yamaoka et al., 2004). JAKs activity malfunction has been reported to be associated with a variety of pathogeneses such as hematopoietic dysfunction, immunodeficiency, autoimmune disorders, allergic, inflammatory diseases, and myeloproliferative disorders viz. rheumatoid arthritis, alopecia areata, ankylosing spondylitis, psoriasis, psoriatic arthritis, Crohn’s disease, systemic lupus arthritis, COVID-19, etc. (Damsky et al., 2021). Dysregulation of JAKs activity occurs by multiple mechanisms, viz. gene translocation, activating or inactivating point mutation, somatic mutation of the receptor, and regulator of JAKs signaling. Activating mutation of the JAK2- JH2 domain, V617F, has been reported by multiple groups associated with polycythemia vera, acute lymphoblastic leukemia, and other myeloproliferative neoplasms (O’Sullivan and Harrison, 2017). Similarly, inactivating mutation of JAK3 and TYK2 are associated with immunodeficiency syndrome like X-linked severe combined immunodeficiency (X-SCID) variant (Russell et al., 1995; Ghoreschi et al., 2009a). The linkage of dysregulated JAK activity and multiple pathologies has gained much attention of global researchers to develop JAK specific inhibitors as targeted therapy or as immunosuppressants.

### 1.2 JAK-STAT signaling pathway

JAKs’ activity depends on their selective interaction with particular cognate receptors. JAKs are localized in the cytosol, endosomes, and near the cell membrane, along with their cognate receptors. However, some reports have also suggested their nuclear localization (Lobie et al., 1996). JAKs are constitutively associated with cytokine receptor activity. Since cytokine receptors lack an intrinsic protein kinase domain, they depend entirely on the enzymatic activities of JAKs attached to an intracytoplasmic portion of cytokine receptors for initial signaling steps (Bousoik and Montazeri Aliabadi, 2018).

Janus Kinase-Signal transducer and activator of transcription (JAK-STAT) signaling pathway is an intracellular signal transduction pathway involved in an array of cytokines, growth factors, and hormones. The JAK-STAT signaling pathway is reported to play a key role in cancer cells’ development, proliferation, differentiation, and survival (Harrison, 2012). The JAK-STAT pathway begins with the binding of ligands to the specific receptor on the cell surface.

After ligand attachment to the receptor, receptor subunits are dimerized to bring together two inactive JAKs intracellularly for transphosphorylation and activation (Figure 1B). Once phosphorylated, JAKs function as dimers (homodimers or heterodimers) and in turn phosphorylates tyrosine residues of the cytoplasmic tail of receptors leading to conformational changes to create docking sites for recruitment of STAT proteins which bind *via* their SH2 domain. Once STAT protein binds with receptor tyrosine residues, JAKs phosphorylate the tyrosine residue of STATs, which induces the dimerization of two STAT monomers (Benveniste et al., 2014). STAT interacting protein (StIP), associated with JAK-STAT, acts as a scaffold to facilitate the phosphorylation of unphosphorylated STAT proteins. Subsequently, the dimerized STAT complex (homodimers or heterodimers) translocates to the cell nucleus *via* importin α-5 dependent Ran nuclear import pathway and attaches to its recognized DNA sequence and initiate transcription of a specific gene to upregulate the expression of cytokines or other components of immune/inflammatory pathways involving positive-feedback mechanism (Shuai and Liu, 2003). In mammals, only seven STAT transcription factors are involved in a wide variety of downstream signaling cascades, namely, STAT1-4, STAT5A, STAT5B, and STAT6 (Murray, 2007).

The JAK-STAT pathway is ubiquitous in the application, but only seven STAT proteins help signal hundreds of cytokine and growth factors. The specificity in targeting gene transcription is achieved by groups of cytokines associating with a specific STAT molecule. STAT3 protein enhances cancer cell proliferation, migration, and survival (Costantino and Barlocco, 2008). Similarly, STAT1 is a key transcription factor involved in the expression of executioner caspases of apoptosis (Sironi and Ouchi, 2004). A defined set of stimuli and receptors are required for a specific member of the STAT family. Other than JAKs, other pathways have been shown to activate STAT proteins; for example, the Src family of kinases has been reported to promote the transcription of VEGF and IL-8 (Trevino et al., 2006). Similarly, activation of STAT3 and STAT1 *via* EGFR and PDGF has also been reported (Zhong et al., 1994).

Due to the ubiquitous use of the JAK-STAT pathway in cytokines, interferons, and growth factor-associated signal transduction and gene transcription, disruption or mutation in the JAK-STAT pathway may result in cell growth dysregulation or immunological disorders (Ghoreschi et al., 2009a). The suppressor of cytokine signaling (SOCS) family of negative regulators regulates JAK-STAT through a negative feedback mechanism by inactivating JAKs proteins and preventing access of STATs to receptor binding site and blocking signaling proteins’ access to the proteasome (Liau et al., 2018). There are eight SOCS proteins, including SOCS1-7 and CIS. The SOCS protein has two domains; one acts as E3 ubiquitin ligase to tag the signaling molecules for the degradation pathway, and another degrades proteins directly or acts as JAK’s pseudo-substrate and inhibits the pathway by obstruction (Durham et al., 2019). The second protein group negatively regulates JAK-STAT pathway functions is protein tyrosine phosphatases (PTPs). The PTPs such as CD45, SHP1, and SHP2 dephosphorylate STAT inhibit their activity and the JAK-STAT pathway (Xu and Qu, 2008). Similarly, the third protein group includes the family of transcription regulators, Protein inhibitors of activated STAT (PIAS). The PIAS family of transcription regulators includes four members such as PIAS1, PIAS2 (PIASX), PIAS3, and PIAS4 (PIASy), which express constitutively in mammals. PIAS interact with activated STAT dimers only and regulate transduction by blocking the binding of STATs to specific DNA sequences (Seif et al., 2017b; Niu et al., 2018). The JAK-STAT pathway is an important signal transduction pathway, considered to play a pivotal role in various disease developments, including cancers (Xin et al., 2020).

## 2 Janus kinase inhibitors (Jakinibs)

The JAK inhibition as the therapeutic approach was first reported in 2003 (Changelian et al., 2003) for the treatment of allogeneic transplants, and almost a decade later, two small molecules Jakinibs, ruxolitinib (2011) and tofacitinib (2012) were approved by the United States Food and Drug Administration (FDA) for the treatment of, MPNs and RA, respectively (Damsky and King, 2017). Subsequently, numerous compounds have been developed and are either being under investigation or approved for a wide variety of autoimmune, autoinflammatory, and cancerous indications, including rheumatoid arthritis, juvenile idiopathic arthritis, psoriatic arthritis, myeloproliferative neoplasms, inflammatory bowel disease (Crohn’s disease and ulcerative colitis), atopic dermatitis, graft-versus-host disease (GVHD) and ankylosing spondylitis. Other conditions being explored include vitiligo, alopecia areata, psoriasis, systemic *lupus erythematosus*, polymyalgia rheumatica, uveitis, scleritis, scleroderma, Sjogren’s syndrome, Takayasu arteritis, giant cell arteritis, hidradenitis suppurativa (Gadina et al., 2019).

Jakinibs are non-immunogenic small molecules that block the critical role of JAKs protein in innate and adaptive immune responses (Gadina et al., 2020). The Jakinibs are usually categorized as first-generation and newer/next-generation Jakinibs. The first-generation Jakinibs act by competitive inhibition by non-covalently interacting with the ATP binding site of the JH1 kinase domain. The ATP-binding site in the JH1 kinase domain is highly conserved among JAKs, resulting in first-generation Jakinibs targeting more than one JAK member both *in vitro* and *in vivo* (Ferrao and Lupardus, 2017). Although these molecules are generally safe and effective, these Jakinibs do not demonstrate high specificity, exhibiting inhibition against more than one Jak member and associated multiple pathways, which explain both the off-target effects and adverse effects (Clark et al., 2014). Consequently, multiple next-generation Jakinibs have been developed with reasonable specificity and selectivity to JAKs and other tyrosine kinase families. These molecules also act in an ATP-competitive manner through non-covalent interaction. Nevertheless, some Jakinibs target the JH2 pseudo-kinase domain of JAK (Deucravacitinib) or act as a covalent inhibitor of JAKs (Ritlecitinib) (Casimiro-Garcia et al., 2018; Burke et al., 2019). These next-generation Jakinibs are expected to increase specificity and reduce adverse events.

## 3 Globally approved Jakinibs

**FIGURE 2:**
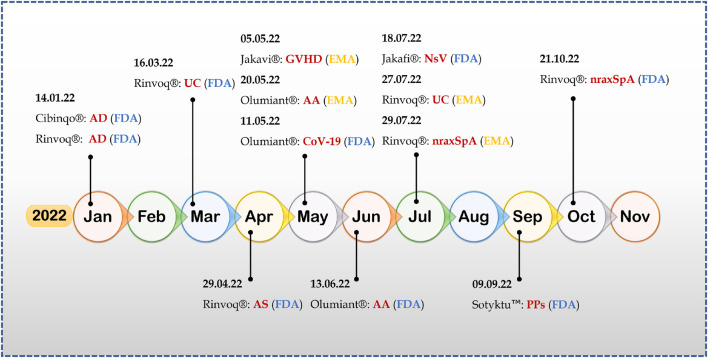
2022-A year of success for Jakinibs (Abbreviations- AD, atopic dermatitis; UC, ulcerative colitis; AS, ankylosing spondylitis; GVHD, graft-versus-host disease; AA, alopecia areata; CoV-19, COVID-19; NsV, non-segmental vitiligo; nraxSpA, non-radiographic axial spondyloarthritis; PPs, plaque psoriasis).

Many Jakinibs have been developed to this date; of them, twelve Jakinibs have been approved by various regulatory agencies for use in multiple immune-inflammatory conditions such as rheumatoid arthritis, psoriatic arthritis, ankylosing spondylitis, juvenile idiopathic arthritis, GVHD, atopic dermatitis, ulcerative colitis, polycythemia vera, myeloproliferative neoplasms, and COVID-19 in humans and atopic dermatitis in canines (Table 1). Figure 2 demonstrates the recent approvals of novel Jakinibs for various disease conditions.

**TABLE 1:** Globally approved Jakinibs for varied therapeutic indications.

S. No.	Jakinib	Targets	Current Indications	Year	Clinical trial identifier^a^ ^b^
1	Ruxolitinib	JAK1, JAK2, JAK2V617F	MF	2011 (FDA)	NCT00952289
				2012 (EMA)	NCT00934544
			PCV	2014 (FDA), 2015 (EMA)	NCT01243944 NCT02038036
			a/cGVHD	2019, 2021 (FDA)	NCT02913261
				2022 (EMA)	NCT03112603
	*Jakafi®; Incyte, Jakavi®; Novartis, Opzelura®; Incyte*				NCT03147742
			AD	2021 (FDA)	NCT03745638
					NCT03745651
			NsV	2022 (FDA)	NCT04052425
					NCT04057573
2	Tofacitinib	JAK1, JAK2, JAK3	RA	2012 (FDA)	NCT00856544
	*Xeljanz®; Pfizer*			2013 (MHLW, Swissmedic)	NCT00847613
				2017 (CFDA)	NCT00814307
				2017 (EMA)	NCT01039688
			PsA	2017 (FDA)	NCT01877668
					NCT01882439
				2018 (EMA)	NCT01976364
			UC	2018 (FDA, EMA)	NCT01465763
					NCT01458951
					NCT01458574
			JIA	2020 (FDA)	NCT02592434
				2021 (EMA)	
			AS	2021 (FDA, EMA)	NCT03502616
3	Oclacitinib	JAK1	Canine AD	2013 (FDA)	NA
	*Apoquel®; Zoetis*				
4	Baricitinib	JAK1, JAK2	RA	2017 (EMA, MHLW)	NCT01721044
				2018 (FDA)	NCT02265705
				2019 (TGA)	NCT01710358
			AD	2020 (EMA)	NCT03334396
					NCT03334422
	*Olumiant®*; *Eli lily and Incyte*		COVID19	2022 (FDA)	NCT04401579
					NCT04421027
			AA	2022 (FDA, EMA)	NCT03570749
					NCT03899259
5	Peficitinib	JAK1, JAK2, JAK3, TYK2	RA	2019 (MHLW)	NCT02308163
	*Smyraf®; Astellas Pharma*				NCT02305849
6	Fedratinib	JAK2, JAK2V617F, FLT3	MF	2019 (FDA)	NCT01437787
	*Inrebic®; Bristol Myers Squibb*			2021 (EMA)	NCT015233171
7	Upadacitinib	JAK1	RA	2019 (FDA)	NCT02706873
					NCT02706951
					NCT02629159
					NCT02675426
				2021 (EMA)	NCT02706847
			PsA	2021 (EMA, FDA)	NCT03104400
					NCT03104374
			AD	2021 (EMA)	NCT03569293
				2022 (FDA)	NCT03607422
					NCT03568318
	*Rinvoq®; AbbVie*		UC	2022 (FDA, EMA)	NCT03006068
					NCT03653026
					NCT02819635
			AS	2021 (EMA)	NCT03178487
				2022 (FDA)	NCT04169373
			nr-axSpA	2022 (EMA, FDA)	NCT04169373
8	Delgocitinib	JAK1, JAK2, JAK3, TYK2	AD	2020 (MHLW)	NCT03826901
	*Corectim®; Japan Tobacco*				NCT03725722
9	Filgotinib	JAK1	RA	2020 (EMA, MHLW)	NCT02889796
					NCT01888874
	*Jyseleca®; Galapagos NV and Gilead Sciences*		UC	2021 (EMA)	NCT02914522
10	Abrocitinib	JAK1, JAK2	AD	2021 (EMA)	NCT03349060
					NCT03575871
					NCT03720470
	*Cibinqo®; Pfizer*			2022 (FDA)	NCT03627767
					NCT03796676
					NCT04345367
11	Pacritinib	JAK2, JAK2V617F, FLT3	MF	2022 (FDA)	NCT02055781
	*Vonjo®; CTI BioPharma Corp.*				
12	Deucravacitinib	TYK2	PPs	2022 (FDA)	NCT03624127
	*Sotyktu™; Bristol Myers Squibb*				NCT03611751

^a^ Information as of December 2022.

^b^ Information assessed from ClinicalTrials.gov, CFDA, China food and drug administration; Swissmedic, the Swiss agency for therapeutic products; TGA, therapeutic goods administration, Australia; MHLW, ministry of health, labor and welfare, Japan; MF, myelofibrosis; PCV, polycythemia vera; a/cGVHD, acute/chronic graft-versus-host-disease; AD, atopic dermatitis; NsV, nonsegmental vitiligo; RA, rheumatoid arthritis; PsA, psoriatic arthritis; UC, ulcerative colitis; JIA, Juvenile idiopathic arthritis; AS, ankylosing spondylitis; COVID-19, coronavirus disease-2019; AA, alopecia areata; nr-axSpA, non-radiographic axial spondyloarthritis; PPs, plaque psoriasis; NA, not applicable.

Upadacitinib has had a scintillating performance this year; till writing this paper, upadacitinib (Rinvoq^®^; AbbVie) has received four FDA approvals and two EMA approvals in a single year for therapeutic indications like atopic dermatitis, ulcerative colitis, ankylosing spondylitis, and non-radiographic axial spondyloarthritis (Shawky et al., 2022). It is an FDA-approved treatment for rheumatoid arthritis (2019) and psoriatic arthritis (2021) (Duggan and Keam, 2019; Funk et al., 2022), making upadacitinib an FDA-approved treatment for six autoimmune and inflammatory disorders.

Deaucravacitinib is the first deuterated derivative and selective allosteric TYK tyrosine kinase Jakinib approved for any therapeutic indication, adding new dimensions to the field of Jakinib drug development and discovery (Thaçi et al., 2022). Furthermore, Deucravacitinib, with its unique mechanism of action, approval came without any black box warning, and it is the first orally dosed treatment approved for moderate-to-severe plaque psoriasis in the last 10 years. It is also being studied for other autoimmune and inflammatory indications, including ulcerative colitis (NCT03934216), Crohn’s disease (NCT03599622), psoriatic arthritis NCT03881059, and systemic *lupus erythematosus* (NCT03920267).

Another deuterated jakinib is deuruxolitinib (CTP-543) which is reported to be a deuterated form of ruxolitinib, developed by Concert Pharmaceuticals, Inc., NY, United States, using its DCE Platform^®^ (deuterated chemical entity platform) (King et al., 2022). Deuruxolitinib was granted FDA breakthrough therapy designation and fast-track designation for treating adult patients with moderate-to-severe alopecia areata. Recently sponsoring company has declared topline results from two pivotal phase III clinical trials in alopecia areata, namely, THRIVE-AA1, and THRIVE-AA2, and it is seeking FDA approval for the treatment of alopecia areata (Concert Pharmaceuticals, 2022).

Alopecia areata is an autoimmune skin disease characterized by unpredictable non-scarring hair loss of the scalp and body, affecting up to 2% of the general population of all ages and ethnicity (Sterkens et al., 2021). No FDA-approved systemic treatment is available for alopecia areata treatment for an extended period. A current understanding of the pathogenesis of alopecia areata led to the focus of Jakinibs toward alopecia areata (Zhou et al., 2021). This year, baricitinib became the first systemic disease treatment for adults with severe alopecia areata, approved by the FDA and EMA (US FDA, 2022). Furthermore, Jakinibs became the first in disease treatment for another dermatological chronic autoimmune disease, namely, non-segmental vitiligo, characterized by skin depigmentation. Recently, FDA approved a 1.5% ruxolitinib cream (Opzelura™, Incyte) as the topical treatment for adults and non-immunocompromised pediatric patients (12 years of age and older). Opzelura™ is the only topical formulation of Jakinib approved in the United States, that too for two therapeutic indications viz. non-segmental vitiligo and atopic arthritis. Almost all FDA or EMA-approved Jakinibs are formulated as solid dosage forms, either tablets or capsules, and only two Jakinibs, including ruxolitinib and delgocitinib, are available as cream and ointment, respectively. Currently, tofacitinib is another Jakinib which under investigation in various phase II clinical trials in the form of 1%–2% ointment for the treatment of alopecia areata (NCT02812342), atopic dermatitis (NCT05487963) and psoriasis (NCT01831466).

Rheumatoid arthritis: Jakinib, as a class, has been associated with enabling the treatment of rheumatoid arthritis to enter a new phase (Nash et al., 2022). Owing to the putative benefit of Jakinibs, even in treating patients who inadequately responded to treatment with methotrexate and/or bDMARDs, five Jakinibs, including filgotinib, upadacitinib, tofacitinib, peficitinib, and baricitinib are available as approved therapeutics in market.

Similarly, for Atopic dermatitis treatment in humans and canines, six Jakinibs include, ruxolitinib, oclacitinib, baricitinib, upadacitinib, delgocitinib, and abrocitinib have been approved worldwide. Among all Jakinibs approved to date, upadacitinib, ruxolitinib, and tofacitinib are the most successful molecules approved for five to six different therapeutic indications.

Myelofibrosis: It is a debilitating clonal hematological malignancy characterized by debilitating constitutional symptoms such as fever, night sweats, weight loss, splenomegaly, bone marrow fibrosis, and cytopenia, with a propensity to transform into acute leukemia due to its origin at the level of the hematopoietic stem cells. (Tremblay and Mascarenhas, 2021). Jakinibs are the only available therapy for myelofibrosis, and currently, there are three FDA-approved Jakinibs ruxolitinib, fedratinib, and the recent one, pacritinib (Lamb, 2022). Ruxolitinib is a JAK1 and JAK2 inhibitor, while fedratinib and pacritinib are JAK2 selective inhibitors with dual FLT3 inhibitory activity (Han et al., 2022). Several selective and non-selective Jakinibs, including gandotinib, ilginatinib, jaktinib, lestaurtinib, momelotinib, and itacitinib, are being investigated for their efficacy in MPNs.

Inflammatory bowel disease: Jakinibs are the most recent therapeutic agents for managing chronic inflammatory bowel diseases such as ulcerative colitis and Crohn’s disease, exhibiting better pharmacokinetic and lack of immunogenicity compared to other biological counterparts (TNF inhibitors) (Hernandez-Rocha and Vande Casteele, 2020). Available Jakinibs for the management of moderate to severe ulcerative colitis include non-selective tofacitinib (FDA) and selective JAK1 inhibitors filgotinib (EMA) and upadacitinib (FDA). However, there are no Jakinibs available approved for the treatment of Crohn’s disease. Researchers are more focused on developing JAK1 selective next-generation Jakinibs with gut-restrictive, systemic sparing agents for treating ulcerative colitis and Crohn’s disease (Spiewak and Patel, 2022). Other next-generation inhibitors under investigation include ivarmacitinib, ritlecitinib, deucravacitinib, ropsacitinib, and izencitinib.

Axial spondylarthritis is a chronic inflammatory disease of the axial skeleton, encompassing two clinical stages: non-radiographic axial spondylarthritis (not detected by X-ray) and active ankylosing spondylitis (Poddubnyy, 2013). Jakinibs represent a new class of therapy that has shown efficacy in treating both subclasses of axial spondylarthritis, i.e., ankylosing spondylitis and non-radiographic axial spondylarthritis. However, there are safety concerns associated with Jakinibs compared to mainstream treatment, including TNF blockers (Akkoc and Khan, 2021). Presently, upadacitinib is the first and only jakinib approved to treat patients with axial spondylarthritis (ankylosing spondylitis and non-radiographic axial spondylarthritis), covering the full spectrum of disease. Tofacitinib is another drug approved for adults with active ankylosing spondylitis only. Both agents represent the alternative therapy for patients with inadequate response or intolerance to TNF blocker therapy.

Similarly, upadacitinib and tofacitinib are the approved choice of treatment for moderate to severe psoriatic arthritis. Upadacitinib has become an alternative therapy for multiple inflammatory indications such as moderate-to-severe rheumatoid arthritis, ankylosing spondylitis, non-radiographic axial spondylarthritis, ulcerative colitis, psoriatic arthritis, and atopic dermatitis, whose patients either had an inadequate response or intolerance to one or more TNF blockers or other systemic therapy.

## 4 Jakinibs and COVID-19

The coronavirus-19 (COVID-19) disease is a respiratory infection caused by SARS-CoV-2, reported first time in China in late 2019 (Zhou et al., 2020). The common symptoms include cold, dry cough followed by sputum, shortness of breath, and dyspnea. When respiratory failure progresses, the disease worsening appears as acute respiratory distress syndrome (ARDS) and cytokine storm leading to hyperinflammation and even death (Zhang et al., 2022). SARS-CoV-2 infection induces cytokine release syndrome (CRS), characterized by elevated pro-inflammatory cytokine generation and secretion, including IL-1, IL-2, IL-6, IL-7, IL-8, IL-10, TNF-α, GCS-F, INF-γ inducible protein 10, MCP-1, and MIP-1α (Mehta P. et al., 2020; Russell et al., 2020). The CRS is implicated in the immunopathogenesis of various disease complications like ARDS and multi-organ failure, including acute lung injury, acute kidney injury, and cardiac injury, even leading to death in COVID-19 patients (Mehta Y. et al., 2020; Yang et al., 2020). The key prognosis markers are elevated serum levels of IL-6 and IFN-gamma (Aliyu et al., 2022). The high mortality rate has been linked with pulmonary embolism and cerebral thromboembolism triggered by vascular disorders attributed to hyperinflammation induced by cytokine storm and multisystem failures, including respiratory failure rendered by microthrombi-induced capillary congestion in alveolar disorders (Tanaka, 2023). In the following sections, we have discussed the role of JAK-STAT signaling in COVID-19 pathophysiology and recent progress in repurposing Jakinibs in the novel clinical setting that is COVID-19.

### 4.1 JAK-STAT signaling in COVID-19 pathogenesis

Several kinase pathways have been implicated in the pathogenesis of COVID-19, including mitogen-activated protein kinases (MAPK)- extracellular signal-regulated kinases (ERK), Janus kinases (JAKs), phosphoinositide 3-kinase (PI3K), and G protein-coupled receptor kinases (GRKs) in regulating the activation, expression, and administration of chemokine receptors, which mediates the various downstream signaling associated with regulation of the immune system and other diverse pathological conditions (Tripathi and Poluri, 2020). JAK-STAT signaling has been reported to play an essential role in SARS-CoV-2-induced hyperinflammation and cytokine release syndrome, contributing to the pathophysiology of ARDS and multisystem failures in affected patients (Kumar and Al Khodor, 2020).

#### 4.1.1 Viral entry and JAK-STAT pathway

Like other viruses, SARS-CoV-2 includes four step life cycle: 1. Viral entry, 2. Viral replication, 3. Viral assembly, and 4. Viral release. SARS-CoV-2 primarily infects alveolar type 2 endothelial cells in the respiratory system with spike 1 protein by binding to angiotensin-converting enzyme 2 (ACE2) receptors and invades alveolar epithelial cells and induces ARDS (Malekinejad et al., 2022). Overexpression of ACE-2 is responsible for viral entry into a variety of target cells. Other than alveolar epithelial cells, many cells express ACE2, including renal epithelial cells, endothelial cells, cardiac myocytes, and immune cells such as macrophages and monocytes in the spleen and lymph nodes. ACE2-dependent viral entry is regulated by two numb associated kinases (NAK), namely, AK1 adaptor protein-associated kinase 1 (AAK1) and cyclin G-associated kinase (GAK), which enables clathrin-mediated receptor endocytosis of SARS-CoV-2 (Pillaiyar and Laufer, 2022). Recently, Luo et al. (2021) reported a close correlation between ACE2 and JAK-STAT pathway in the regulation of immune responses, including activation of B lymphocytes, helper T cells 1 (Th1), and the inhibition of CD8^+^ T cells and Foxp3^+^ regulatory T cells (Treg), suggesting the role of overexpressed ACE2 in downstream JAK-STAT signaling to modulate SARS-CoV-2 induced inflammatory responses. In the correlation analysis, JAK2, STAT1, STAT2, STAT4, and STAT5A were upregulated in significant association with ACE2. Similarly, Interferon-alpha 2 is reported to further augment the viral load by upregulating the ACE2 expression in a loop-back mechanism in different cell systems (Wilk et al., 2020). The role of JAK signaling in the transcription and activation of ACE2 in lung epithelium has also been reported in another study (Lee et al., 2020). Recently, Al-Ani et al. (2022) reported the involvement of lung angiotensin II to type 1 receptor (AT1R)-JAK-STAT axis in lipopolysaccharides (LPS)-induced cytokine storm and resulting acute lung injury and coagulopathy corresponding with moderate-to-severe COVID-19 in humans.

#### 4.1.2 Escaping immunity and viral replication

In the early stage of infection, SARS-CoV-2 induces negative regulator proteins SOCS3 to inhibit STAT1/IFN and glycoprotein 130 (gp130)/JAK2-STAT3 signaling to escape antiviral responses and replicate freely (Zizzo et al., 2022). Furthermore, SARS-CoV2 delays innate and adaptive immune response for too long. SARS-CoV-2 infection is characterized by the reduced cell count of four fundamental components of adaptive immunity, such as natural killer cells, B lymphocytes, CD4^+^ T cells, and CD8^+^ T cells (Zheng et al., 2022). Additionally, several investigations have revealed the impaired antiviral response of JAK-STAT pathway-mediated type I interferons due to SARS-CoV-2 virus infection (Walz et al., 2021). One author reported a rare X-chromosome loss-of-function mutation that impairs type I interferon response (Yuan et al., 2020), while another group reported an association between disease severity and type I interferon deficiency in COVID-19 patients, indicating innate immunity dysregulation (van der Made et al., 2020).

#### 4.1.3 Cytokine storm, ARDS, and JAK-STAT pathway

In COVID-19 patients, viral entry stimulates multiple signaling pathways and produces a broad spectrum of clinical manifestations such as acute respiratory distress syndrome (ARDS), multi-organ failure, and even death (Rarani et al., 2022). The primary etiological factor is a systemic immune activation characterized by elevated levels of pro-inflammatory cytokines (IL-2, IL-4, IL-6, IL-7, IL-10, TNF-alpha, IFN-gamma) and chemokines (CCL2, CCL8), also known as cytokine release syndrome or cytokine storm (Khaledi et al., 2022). One of several signaling pathways is the JAK-STAT signaling pathway in the immunity and immunopathology of COVID-19 disease (Luo et al., 2020). JAK-STAT pathway acts as the downstream signaling mechanism for most of the pro-inflammatory cytokines, principally IL-6, to confer immunomodulatory functions in the body. Various studies have proposed the involvement of the IL-6/GM-CSF/JAK-STAT axis in developing COVID-19-associated cytokine release syndrome. Moreover, JAK-STAT signaling has been implicated in SARS-CoV-2-induced inflammation by recruiting macrophages, monocytes, neutrophils, natural killer cells, lymphocytes, and dendritic cells towards the cytokine storm and eventual manifestation of ARDS and even death (Satarker et al., 2021). JAK1,2 has also been implicated in local complement hyperactivation due to SARS-CoV-2 infection (Yan et al., 2021). Similarly, JAK1,3 and STAT1,3,5 mediate the IL2 signaling to enhance the cytotoxicity of natural killer cells. IL-6. JAK1 also mediates the IL-15-dependent NK cell development, growth, and functioning (Gotthardt et al., 2019). The severity of COVID-19 is related to JAK1-STAT1 dysregulation and compensatory hyperactivation of STAT3. STAT3 is a key transcription factor for harmful IL-6 that stimulates transforming growth factor-1beta, which plays a crucial role in pulmonary fibrosis in COVID-19 acute lung injury (Matsuyama et al., 2020).

#### 4.1.4 Microvascular and macrovascular complications and multi-organ failure

Several micro- and macrovascular complications have been reported in COVID-19 patients. Cardiovascular, cerebrovascular, renal, and thrombotic issues lead to catastrophic-end organ damage and multisystem organ failure, which drive COVID-19 mortality (Levi et al., 2020). In patients with cardiovascular risk factors such as hypertension, diabetes, and obesity, a higher risk of developing thrombotic complications has been reported (Stefan et al., 2020). SARS-CoV-2 induced cytokine storm and subsequent activation of JAK-STAT pathway in target cells, including endothelial cells and inflammatory cells, is linked with upregulated tissue factors and other thrombotic factors like von Willebrand factor and associated extrinsic coagulation cascade implicated in various macro- and microvascular thrombosis. Additionally, increased JAK3 expression and activity contributed to platelet hyperactivation manifesting as platelet aggregation, P-selectin expression, adhesion, and spreading ability during COVID-19 infection, validating the role of JAK-STAT signaling in driving thrombotic processes and organ dysfunction (Ravid et al., 2022). SARS-CoV-2 infection is also implicated in causing a severe kidney disease called COVID-19-associated nephropathy (COVAN), characterized by the collapse of glomerular capillaries and loss of podocytes. In an *in vitro* examination, Nystrom et al. (2022) demonstrated that podocyte loss and COVAN phenotype resulted from cytokine-induced activation of pathogenic APOL1 protein expression *via* the JAK-STAT pathway and Jakinibs may block this pathogenic process.

### 4.2 Jakinibs for COVID-19 management

**FIGURE 3:**
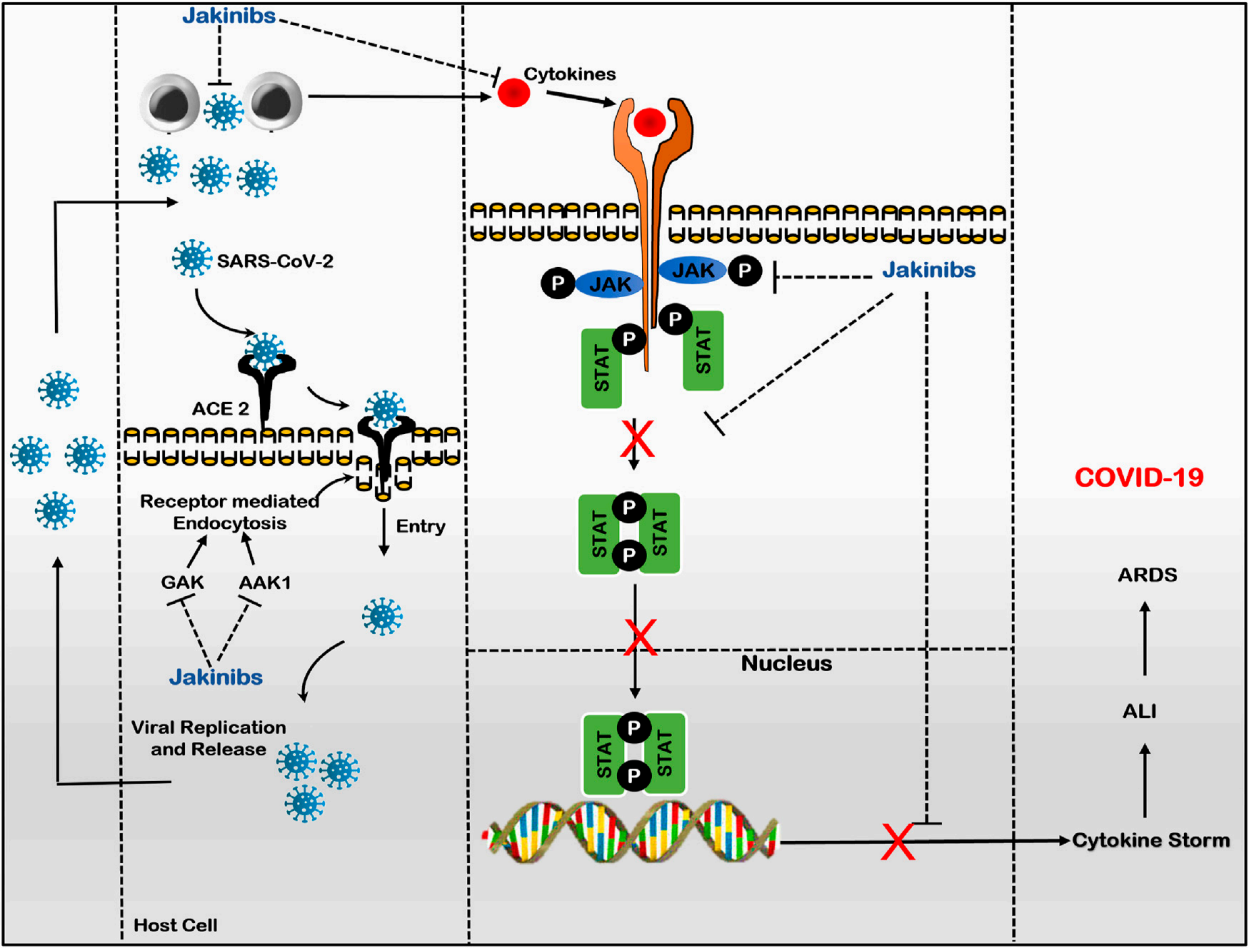
Plausible mechanism of Jakinibs in COVID-19 (Baricitinib as prototype drug): Jakinibs inhibit viral entry and intracellular assembly of virus in target cells by inhibiting AAK1 and GAK-dependent receptor-mediated endocytosis. Furthermore, Jakinibs inhibit various JAKs proteins, suppressing the phosphorylation and nuclear translocation of STAT proteins and downregulating the transcription of cytokine genes. Thus, Jakinibs suppress the release of various pro-inflammatory cytokines (Cytokine storm; IL-6, IL-12, IFN-γ, and GM-CSF) and subsequent immune dysregulation, alleviating hyperinflammation state and ARDS in COVID-19 patients. (Abbreviations- SARS-CoV-2, severe acute respiratory syndrome-coronavirus-2; ACE-2, angiotensin-converting enzyme-2; GAK, cyclin G-associated kinase; AAK1, AK1 adaptor protein-associated kinase 1; JAK, Janus kinase; STAT, signal transducer and activator of transcription; ALI, acute lung injury; ARDS, acute respiratory distress syndrome; COVID-19, Coronovirus-2019 disease).

The Jakinibs have proved to be a promising treatment for treating a diverse group of inflammatory and autoimmune diseases, evolving as a class of drugs with expanded drug selectivity, organ specificity, and therapeutic indications (Lin et al., 2020; Alexander et al., 2021). A recent addition to the therapeutic indications of Jakinibs is COVID-19 disease. In an attempt to manage COVID-19, Jakinibs caught the attention of researchers when baricitinib potential was identified using artificial intelligence in its ability to bind with high affinity to two human numb associated kinases, namely, AP2-associated protein kinase-1 and cyclic G-associated kinase (GAK), which regulates clathrin-mediated endocytosis. Thus, inhibiting SARS-CoV-2 virus entry into host cells and subsequent intracellular replication, assembly, and release of virus particles, indicating the anti-viral potential of Jakinibs (Stebbing et al., 2020; Zizzo et al., 2022). Later on, due to the JAK-STAT pathway’s role in SARS-CoV-2-induced dysregulated hyper-immunity, inflammation, and fibrosis, Jakinibs have gained impetus to be repurposed as COVID-19 patient treatment due to their anti-inflammatory and immunomodulatory activity (Montero et al., 2021). Various Jakinibs were later reported to exert multidirectional anti-cytokine effects by inhibiting downstream intracellular JAK-STAT signaling induced by several pro-inflammatory cytokines, particularly IL-6 and GM-CSF. Jakinib’s effect on cytokine signaling depends on type of JAKs; on binding to JAK1, Jakinibs would broadly inhibit signaling by gp130 family cytokine receptors (e.g., IL-6, IL-27), IFN family cytokine receptors (IFN-gamma); and acting on JAK2 would specifically hinder with GM-CSF, IL-12, IL-23, and IL-6 signals. JAK1 and JAK3 inhibition would more potently interfere with gamma-chain family cytokine receptors (e.g., IL-4, IL-7, IL-9) (Hammarén et al., 2019). Jakinibs inhibiting cytokine release and signaling also alleviate pneumonia and ARDS, cumulating to hyperinflammation and multisystem organ failure (Gajjela and Zhou, 2022). Figure 3 summarizes the plausible mechanism of Jakinibs in COVID-19 disease. Other immunomodulatory agents like monoclonal antibodies and interleukin antagonists usually target one cytokine, whereas Jakinibs may provide the strategic advantage of simultaneously targeting multiple cytokines, including IL-6 and GM-CSF (Rommasi et al., 2022). Herein, we will discuss the clinical status of major Jakinibs approved or under investigation for COVID-19 diseases.

Baricitinib: also known as Olumiant and LY3009104, is a JAK1/2 inhibitor that reversibly inhibits JAKs JH1 tyrosine kinase domains in an active conformation. It also acts as an ATP-competitive inhibitor (Coricello et al., 2020). Baricitinib is efficacious in treating multiple autoimmune and inflammatory diseases, including rheumatoid arthritis, systemic *lupus erythematosus*, juvenile dermatomyositis, and atopic dermatitis (Banerjee et al., 2017). This small molecule Jakinib was generated by modifying the tofacitinib structure, and similar to tofacitinib, baricitinib is mainly used in patients with failed therapies with previous drugs (Taylor, 2019).

Among Jakinibs, Baricitinib is the first jakinib to join the COVID-19 therapeutic arsenal. Recently, FDA has approved baricitinib as the first immunomodulatory drug to treat hospitalized COVID-19 adult patients requiring supplemental oxygen, non-invasive or invasive mechanical ventilation, or ECMO. Baricitinib, combined with remdesivir, has previously got FDA’s EUA status for treating COVID-19 among hospitalized adult and pediatric patients (Rubin, 2022). Currently, baricitinib is being investigated in at least 28 clinical trials for evaluating effectiveness in patients with COVID-19 (Table 2). FDA approval of baricitinib was supported by two phase 3 randomized, double-blind, placebo-controlled trials: ACTT-2 and COV-BARRIER. In ACTT-2 (Adaptive COVID-19 Treatment Trail) trial (NCT04401579), randomized 1,033 hospitalized adult COVID-19 pneumonia patients were to receive baricitinib plus remdesivir (515) or remdesivir alone (518). In the results, the combination treatment with the Jakinib baricitinib and the antiviral drug remdesivir was safe and superior to remdesivir alone in reducing recovery time (7 days vs. 8 days) and accelerating improvement of oxygenation among COVID-19 patients requiring high flow oxygenation or non-invasive mechanical ventilation. Moreover, combination therapy was associated with less frequent severe adverse events than control treatment (Kalil et al., 2021). Similarly, in the COV-BARRIER phase III trial (NCT04421027), 1,525 hospitalized adults with COVID-19 receiving standard-of-care treatment (systemic corticosteroids including dexamethasone and antivirals such as remdesivir) were randomized to baricitinib group (764) and the placebo group (761). The baricitinib plus standard of care group demonstrates lower 28-day all-cause mortality (8%) compared to the placebo plus standard of care group (13%). Similar results appeared in evaluating the 60-day all-cause mortality among the two groups. Although the treatment with baricitinib plus standard of care showed a similar safety profile with reduced mortality in hospitalized COVID-19 patients, there was no difference in the frequencies of serious adverse events, severe infections, and venous thromboembolic events between the two groups (Marconi et al., 2021).

**TABLE 2:** Clinical Trials of Jakinibs with COVID-19 patients.

Jakinibs	Combination	Clinical Trial^a^ ^b^				
		Clinical trial Identifier	Location	Start Date	Phase	outcomes
Baricitinib		NCT04390464	United Kingdom	May 8, 2020	4	Unknown
		NCT04421027	USA	June 12, 2020	3	Completed
		NCT05056558	Bangladesh	October 2021	3	Not yet recruiting
		NCT05074420	USA	December 21, 2021	3	Recruiting
		NCT04340232	USA	April 9, 2020	2/3	Withdrawn (Could not make FDA-required changes)
		NCT04358614	Italy	March 16, 2020	2/3	Completed
		NCT04891133	Austria	May 18, 2021	2/3	Recruiting
		NCT04381936	Multinational	May 11, 2020	2/3	Recruiting
		NCT04393051	Italy	May 20, 2020	2	Unknown
		NCT04346147	Spain	May 7, 2020	2	Active, not recruiting
		NCT04399798	Italy	May 22, 2020	2	Unknown
	Remdesivir	NCT04970719	Bangladesh	July 10, 2021	3	Recruiting
		NCT04693026	Bangladesh	September 10, 2020	3	Recruiting
		NCT04401579	USA	May 8, 2020	3	Completed
		NCT04640168	USA	December 2, 2020	3	Completed
		NCT04321993	Canada	April 17, 2020	2	Recruiting
	Dexamethasone, Ramdesivir	NCT04832880	Italy	April 6, 2021	3	Not yet recruiting
	Lopinavir/Ritonavir	NCT04320277	Italy	May 16, 2020	2/3	Unknown
	Dexamethasone	NCT04890626	Spain	May 18, 2021	3	Recruiting
	Olokizumab	NCT05187793	Russian Federation	July 8, 2021	3	Recruiting
	Hydroxychloroquine	NCT04373044	USA	May 4, 2020	2	Terminated (The study was terminated after the release of results of ACTT-2 (NCT04401579).
	Tocilizumab	NCT05082714	Greece	October 19, 2021	NA	Recruiting
	Tocilizumab, inhaled DNase	NCT05279391	Greece	April 20, 2021	NA	Recruiting
Ruxolitinib		NCT04377620	USA	May 24, 2020	3	Terminated (Study terminated by sponsor)
		NCT04362137	USA	May 2, 2020	3	Completed
		NCT04348071	USA	July 2021	2/3	Withdrawn (Could not make FDA-required changes)
		NCT04477993	Brazil	August 14, 2020	2/3	Terminated (Low accrual)
		NCT04354714	USA	June 30, 2020	2	Withdrawn (Could not make FDA-required changes)
		NCT04414098	USA	June 1, 2020	2	Unknown
		NCT04403243	Russian Federation	May 8, 2020	2	Completed
		NCT04338958	Germany	April 22, 2020	2	Completed
		NCT04334044	Mexico	September 1, 2020	1/2	Completed
		NCT04359290	Germany	July 1, 2020	2	Completed
		NCT04581954	UK	October 2, 2020	1/2	Recruiting
		NCT04331665	Canada	May 21, 2020	NA	Terminated
	Tocilizumab, Anakinra	NCT04424056	France	September 1, 2020	3	Unknown
	Anakinra	NCT04366232	France	August 19, 2020	2	Terminated (investigator decision)
	Therapeutic Plasma Exchange	NCT04374149	USA	April 30, 2020	2	Completed
	Simvastatin	NCT04348695	Spain	April 12, 2020	2	Completed
Tofacitinib		NCT04412252	USA	June 2, 2020	2	Withdrawn by sponsor
		NCT04415151	USA	October 14, 2020	2	Terminated (enrolment issues)
		NCT04750317	Russian Federation	May 11, 2020	2	Completed
		NCT04469114	Brazil	September 16, 2020	3	Completed
	Hydroxychloroquine	NCT04390061	Italy	June 2020	2	Unknown
	mRNA vaccine	NCT05080218	USA	November 15, 2021	4	Recruiting
Pacritinib		NCT04404361	USA	May 22, 2020	3	Terminated
Nezulcitinib		NCT04350736	United Kingdom	April 23, 2020	1	Completed
		NCT05091723	USA	October 13, 2021	1	Completed
		NCT04402866	Multinational	June 24, 2020	2	Completed

^a^ Information as of December 2022.

^b^ Information assessed from ClinicalTrials.gov.

The efficacy and safety of baricitinib plus standard of care were also evaluated among critically ill hospitalized COVID-19 adult patients receiving invasive mechanical ventilation or ECMO in an exploratory study, randomizing 101 patients into baricitinib (4 mg QD) plus standard of care (51) and placebo plus standard of care (49). Baricitinib showed a significant reduction in both 28-day and 60-day all-cause mortality compared to placebo, consistent with the main phase III study involving less severely ill patients (Ely et al., 2022). The lack of clear-cut efficacy of baricitinib in intubated COVID-19 patients in the ACTT-2 trial prompted another phase III randomized double-blind, placebo-controlled ACTT-4 trial, in which a combination of baricitinib plus remdesivir and dexamethasone plus remdesivir were compared in hospitalized COVID-19 adult patients requiring supplemental oxygen administration by low/high flow, or non-invasive ventilation. In the hospitalized patients, both the groups resulted in similar mechanical ventilation-free survival by day 29 (87% vs. 87.6%). However, the dexamethasone group was associated with more severe or life-threatening grade 3 or 4 adverse events than the baricitinib group (Wolfe et al., 2022). The EULAR, in its recent update in 2021 on the use of immunomodulatory agents as treatment of COVID-19, has suggested the use of baricitinib and tofacitinib in combination with glucocorticoids to treat hospitalized patients with COVID-19 requiring oxygen supplementation, non-invasive ventilation or high-flow oxygen (Alunno et al., 2022). A recent meta-analysis of 12 studies on 3,564 patients has reported using baricitinib as a safe and effective anti-COVID-19 drug candidate with reduced risk of adverse events and better clinical efficacy in the high-dose groups (Lin et al., 2022). In an ongoing large-scale phase III clinical trial (RECOVERY) evaluating multiple possible treatment modalities in hospitalized COVID-19 patients, baricitinib (4 mg QD) demonstrated a significant reduction in the risk of death. However, the proportional reduction in mortality was smaller than the meta-analysis of eight clinical trials. The updated meta-analysis of all nine clinical trials, including the results from the RECOVERY trial (NCT04381936), suggested a one-fifth reduction in mortality of patients hospitalized for COVID-19 with baricitinib or other Jakinibs. (Rate ratio 0·80) (Mehta and Titanji, 2022). Recently, the World Health Organization (WHO) has also recommended baricitinib for severe or critical COVID-19 patients in combination with corticosteroids (Kmietowicz, 2022).

Ruxolitinib: is an ATP-competitive inhibitor of JAK1/2, which targets active conformation of the JH1 kinase domain of JAK1 and JAK2 (Arana Yi et al., 2015). Ruxolitinib, also designated as INCB018424 or INC424, was the first jakinib approved by the FDA for treating the patient with intermediate or high-risk myelofibrosis in November 2011 (Seavey and Dobrzanski, 2012). Later, in December 2014, the FDA approved ruxolitinib for treating PV. In 2019 and 2021, ruxolitinib got approval for treating acute and chronic GVHD, respectively (Mascarenhas and Hoffman, 2012; Raedler, 2015; Yang et al., 2021). Additionally, on 5 May 2022, the European medicines agency (EMA) approved ruxolitinib for treating 12 years or older patients of acute/chronic GVHD, dependent or refractory to corticosteroids or other systemic therapies. The FDA has also approved Ruxolitinib for treating atopic dermatitis as a 0.75% and 1.5% cream formulation (Papp et al., 2021; Shalabi et al., 2022). Ruxolitinib is the first topical formulation approved for repigmentation in vitiligo patients. The FDA has approved ruxolitinib cream (1.5%) for treating non-segmental vitiligo in adult and pediatric patients 12 years and older (Rosmarin et al., 2022).

Similarly, ruxolitinib is under investigation for treating COVID-19 patients. In a case study by Innes et al. (2020), the administration of ruxolitinib at a dose of 10 mg BID resulted in an improvement in respiratory function with a corresponding reduction in the fraction of inspired oxygen (FiO2) in tocilizumab refractory severe COVID-19 patient. Furthermore, Iastrebner et al. (2021) prospectively assessed 102 COVID-19 pneumonia patients treated with ruxolitinib 5 mg BID. The study results revealed a non-significant reduction in ICU admission and mechanical ventilation requirement and a lower mortality rate in critically ill COVID-19 patients (NCT04414098).

Similar results were demonstrated by Cao et al. (2020) in a prospective randomized controlled trial; ruxolitinib at 5 mg BID dose produced no significant difference in the 28-day mortality rate and clinical improvement in severe COVID-19 patients. However, compared to the control group, ruxolitinib treatment was associated with faster clinical improvement, chest computed tomography improvement at day 14, and no change in serious adverse events. Currently, there are 22 clinical studies listed on ClinicalTrials.gov, only six studies have been completed, and an equal number of trials have been terminated or withdrawn (Table 2). Notably, sponsors terminated an interventional phase III clinical trial (RUXCOVID-DEVENT), observing no significant difference in the 28-day mortality rate in ruxolitinib group and placebo group in the ruxolitinib group and placebo group COVID-19-associated acute respiratory distress syndrome patients on mechanical ventilation (Rein et al., 2022). Similarly, in another international, randomized, phase 3 trial (RUXCOVID; NCT04362137) of ruxolitinib vs. placebo, associated with the standard of care in hospitalized COVID-19 patients but not on mechanical ventilation or in ICU, ruxolitinib 5 mg BID did not meet its primary endpoint and demonstrated no benefit in the overall studied population (Han et al., 2022). In phase II non-randomized multicenter clinical trial on sixteen COVID-19-associated ARDS and hyperinflammation patients (NCT04359290), a ruxolitinib treatment was shown to be a well-tolerated and efficacious and feasible therapy for COVID-19-induced ARDS patients requiring invasive mechanical ventilation (Neubauer et al., 2021). In summary, ruxolitinib is a potential candidate for COVID-19-associated ARDS, but the available evidence is based on limited sample sizes in completed and published clinical trials. Most available information is based mainly on case reports and cohort studies; thus, studies must be made on larger sample sizes to assess its risk/benefit ratio.

Tofacitinib: It also known as CP-690,550, Xeljanz, and tosacitinib, is a first-generation JAK 1/2/3 inhibitor, acts on the active conformation of the JH1 kinase domain of JAK1/2/3 *via* ATP-competitive inhibition. Tofacitinib was initially tried to prevent allograft rejection but was later abandoned due to high dosage and excessive immunosuppression (Webber and Vincenti, 2016; Coricello et al., 2020). In 2012, tofacitinib got FDA approval for rheumatoid arthritis; since then, it has gained regulatory approval for four other indications, including adult and juvenile psoriatic arthritis (2017), ulcerative colitis (2018), polyarticular-juvenile idiopathic arthritis (2020), and ankylosing spondylitis (2021) (Caporali and Zavaglia, 2019; Mohanakrishnan et al., 2022; Shawky et al., 2022).

Currently, six clinical trials are either completed or under investigation for assessing the safety and efficacy of tofacitinib in COVID-19 patients (Table 2). Maslennikov et al. (2021) reported a lower mortality rate (16.6% vs. 40.0%) and incidence of admission to the ICU (15.6% vs. 50.0%) in 32 patients affected by COVID-19 associated CRS (CRP>150 mg/L) and treated with tofacitinib (10 mg BID on the first day, then 5 mg BID for next 4 days) compared to 30 patients without prior treatment with any anti-cytokine drugs. Tofacitinib treatment also reduced hyperinflammation and the volume of affected lungs with a significant increase in oxygen saturation. In a recently concluded, multicenter, randomized, double-blind clinical trial of tofacitinib in hospitalized COVID-19 pneumonia patients (STOP-COVID; NCT04469114), Guimarães et al. (2021) randomized a total of 289 patients in a 1:1 ratio to receive either tofacitinib (10 mg BID) or placebo (BID) for up to 14-day or until hospital discharge. 89.3% of patients were on glucocorticoid therapy as a standard of care. The results revealed that tofacitinib at a dose of 10 mg BID could lower the mortality or respiratory failure than placebo through day 28. Furthermore, tofacitinib and glucocorticoid therapy lead to a lower risk of clinical events among hospitalized COVID-19 pneumonia patients than placebo. EULAR, in its recent update, has suggested using tofacitinib in combination with glucocorticoids as immunomodulatory therapy among critically ill or severe COVID-19 patients requiring oxygen therapy for reduced disease progression and mortality (Alunno et al., 2022). Similar findings have been reported by multiple observational studies and randomized controlled trials, suggesting the use of tofacitinib alone or in combination with dexamethasone to improve survival odds in severe and critical COVID-19 patients (Singh P. K. et al., 2021; Hayek et al., 2021; Panda et al., 2021; Shrestha et al., 2021; Ferrarini et al., 2022; Kodali et al., 2022; Murugesan et al., 2022). Based on available evidence, tofacitinib is a valid therapeutic option for COVID-19; however, more studies are needed compared to clinical trials, as the results are reported mainly from case studies.

Nezulcitinib: It is also known as TD-903, is a nasally inhalable lung-selective pan-JAK inhibitor developed for treating acute lung injury associated with COVID-19 (Pfeifer et al., 2021; Zhang et al., 2021). Notably, nezulcitinib has been evaluated in three clinical trials (NCT04350736, NCT04402866, and NCT05091723) (Table 2). Results of two studies have been published, wherein in the phase I study, safety and efficacy were assessed in healthy participants. Nezulcitinib was well tolerated as a single inhalable dose of up to 10 mg without serious or severe adverse events (Pfeifer et al., 2021). Similarly, in part 1 of the phase II clinical trial randomizing, 25 patients already received dexamethasone (92%)/remdesivir (12%) as the standard of care. Once inhaled daily, nezulcitinib demonstrated improvement in respiratory failure-free survival and SaO2/FIO2 ratio at day 28 and shorter mean time to hospital discharge in severe COVID-19 patients versus placebo.

Additionally, nezulcitinib treatment was associated with lower mortality (5% vs. 33%) compared to placebo. There was no incidence of severe adverse events reported. Interestingly, pan-Jak inhibitor nezulcitinib has shown the potential to target cytokine-driven pulmonary inflammation in severe COVID-19 patients in combination with dexamethasone. Based on the positive results in the part 1 study, nezulcitinib 3 mg is being studied in part 2 of this phase II trial, including a larger population, double-blind, placebo-controlled parallel group in hospitalized patients of COVID-19 requiring oxygen supplementation (Singh D. et al., 2021).

Pacritinib: It is also known as SB-1518 and VONJO™, is a multi-kinase selective inhibitor of JAK2, JAK2V617F, fms-like receptor tyrosine kinase 3 (FLT3), colony-stimulating factor 1 receptor (CSF1R), and IRAK-1 with IC_50_ < 50 nM (Singer et al., 2016). Pacritinib was developed and tested for its activity in treating myelofibrosis (Hart et al., 2011; William et al., 2011; Beauverd et al., 2015). Pacritinib inhibits JAK2 by preferentially binding to its activated form in an ATP-competitive manner (Hart et al., 2011).

In an *in vitro* study, pacritinib was shown to inhibit GU-rich single-stranded RNA derived from SARS-CoV-2 induced IRAK1 activation and TLR8-dependent pro-inflammatory cytokines release such as IL-1β, TNF, and IL-6 (Campbell et al., 2022). Clinically, pacritinib was analyzed in a randomized, double-blind, placebo-controlled, multicenter phase III study (PREVENT; NCT04404361) to compare pacritinib plus standard of care vs. placebo plus standard of care in hospitalized severe COVID-19 patients with or without cancer (Table 2). The primary endpoint of the study was the proportion of the patients who progress to invasive mechanical ventilation (IMV) and/or ECMO or died through day-28. Unfortunately, the study was terminated by the sponsors, as the pacritinib did not demonstrate significant improvement in the primary endpoint of progression to IMV and/or ECMO or death in treating serious or severe COVID-19 patients by Day 28.

Other Jakinibs, like fedratinib and jaktinib, have been reported to be promising candidates in COVID-19 management. An *in vitro* study by Wu and Yang, (2020) suggested the promising potential of selective JAK2 inhibitor fedratinib in suppressing TH17-associated cytokine production (IL-17, IL-22, GM-CSF) during the management of COVID-19 and other related viruses. Similarly, Meng et al. (2020) have highlighted the potential of pan-JAK inhibitor jaktinib hydrochloride for treating COVID-19 patients, given the role of jaktinib in preventing JAK-dependent cytokine-induced immune activation and AAK1 and GAK-mediated viral entry and subsequent proliferation (Figure 3).

Theoretically, combination therapy is advantageous compared to monotherapy concerning decreased drug resistance and reduced risks of adverse events (Akbarzadeh-Khiavi et al., 2022). In this context, in a double-blind, randomized controlled trial, baricitinib combined with the anti-viral drug remdesivir was superior to remdesivir monotherapy in reducing recovery time and improving clinical outcomes in hospitalized COVID-19 patients receiving oxygen or non-invasive ventilation. Another observational cohort study demonstrated remarkable improvement in pulmonary function with baricitinib plus corticosteroid therapy compared to corticosteroid therapy alone (Rodriguez-Garcia et al., 2021). Jakinibs are reported to exhibit safety comparable to multiple biologics, sometimes more efficacious. However, despite the several advantages of Jakinibs over biologics, such as multi-target blocking and ease of administration, there are serious concerns related to the management and treatment of serious adverse effects, including cardiovascular disorders, thrombotic complications, malignant tumors and risk of deaths, which are more significant than the use of biologics (Tanaka et al., 2022).

Multiple clinical trials are either in recruiting stage or under investigation related to the combination of baricitinib with different antiviral and immunomodulatory agents, including remdesivir, ritonavir, dexamethasone, and tocilizumab, etc., as shown in Table 2.

### 4.3 Limitations

In general, due to their role in the blockage of multiple cytokines involved in many physiological functions such as host defense, hematopoiesis, metabolism, cell growth, and differentiation, Jakinibs have been linked to multisystem impacts. A variety of side effects have been reported to be linked with jakinib treatment, such as opportunistic infections, anemia, venous thromboembolism (VTE), deep vein thrombosis (DVT), pulmonary embolism, hyperlipidemia, and malignancy risks (Alexander et al., 2021).

Similar micro- and macrovascular complications manifest in the severe cases of COVID-19 due to SARS-CoV-2 mediated dysregulation of immune functions and upregulation of tissue factors and other thrombotic factors like von Willebrand factor augmenting extrinsic coagulation cascade (Ravid et al., 2022). Moreover, Overexpressed JAK3 downstream to MAPK signaling is reported to mediate platelet hyperactivation. Hence, using JAK1-2 inhibitors may not appear as a potent therapeutic option. Furthermore, Opportunistic infections due to pathogenic bacteria and viruses are the primary concern associated with pan-JAK inhibitors, as JAK1 is involved in cell signaling that regulates type I-III interferons in host immunity to produce antibacterial and antiviral responses. Using pan-JAK inhibitors might interfere with type I and type II antiviral and antibacterial responses, allowing opportunistic infection (Kalil et al., 2021). Various studies have documented the increased incidence of secondary infections (Luo et al., 2020). Urinary tract infections were the most common adverse events in patients treated with ruxolitinib, while bacterial infections of the upper respiratory tracts were the most significant side effect associated with baricitinib therapy (Pillaiyar and Laufer, 2022). Multiple accumulated data suggest that blocking JAK-STAT signaling by irreversible Jakinib is potentially dangerous due to the risk of severe immunodeficiency; temporary and reversible JAK inhibition may provide safety and efficacy in treating many inflammatory conditions, including COVID-19 (Rommasi et al., 2022).

Due to the non-involvement of JAK2 in type I or type II interferon-related cell signaling, selective JAK2 inhibitors are the preferred treatment choice for suppressing IL-6-GM-CSF-signaling-in COVID-19-associated cytokine storms. Jakinibs have also been reported to increase the risk of herpes virus reactivation, e.g., herpes zoster and herpes simplex (Huang et al., 2022). Epidemiological studies revealed the limitation of baricitinib therapy in patients with an absolute neutrophil count of less than 1 × 10^9^ cells/L or an absolute lymphocyte count of less than 0.5 × 10^9^ cells/L. Furthermore, baricitinib therapy increases anemia incidence in patients with COVID-19 (Praveen et al., 2020). Recently, jakinib treatment was also reported to significantly reduce the humoral response following dual vaccination against SARS-CoV-2 in rheumatoid arthritis patients. The humoral response was further impaired in patients when jakinib was co-administered with the disease-modifying anti-rheumatoid drug (DMARDs), methotrexate (Schäfer et al., 2022).

Despite safety concerns associated with clinical use, various meta-analysis studies have validated and confirmed the safety and efficacy of jakinib treatment of hospitalized COVID-19 patients. Manoharan and Ying, (2022), in a meta-analysis study, analyzed fifteen articles, including randomized controlled trials and non-randomized controlled trials related to the use of baricitinib in the management of COVID-19 patients; the study revealed that baricitinib significantly reduced the mortality rate and disease progression in COVID-19 patients. Similarly, Chen et al. (2021) compared three RCTs of Jakinibs (ruxolitinib, baricitinib, and tofacitinib) in treating hospitalized COVID-19 patients. All three Jakinibs were found to improve the clinical outcomes of COVID patients in the form of lower mortality rate, higher clinical recovery rate, and a lower rate of mechanical ventilation use than control groups. In another meta-analysis of five studies with a total of 1,190 patients, Wijaya et al. (2021) reported a reduced risk of clinical deterioration, clinical improvement, and reduced risk of mortality associated with jakinib (baricitinib and ruxolitinib) use in hospitalized COVID-19 patients.

Regarding safety issues associated with the use of Jakinibs, Chen et al. (2021) and Kramer et al. (2022), in two different meta-analyses, did not find any association between the use of jakinib and a higher risk of adverse events of any grade and secondary infections in COVID-19 patients compared to the control groups. In a recent pooled analysis of seven randomized controlled trials, Jakinib in the treatment group significantly reduced the all-cause 28-day mortality rate (31%) and risk of 14-day mortality (35%) than the control group (Tang et al., 2022). Also, both Jakinibs and COVID-19 are linked with potential thrombotic complications of their own, but to date, there is no existing evidence suggesting increased thrombotic risk associated with Jakinibs use in treating COVID-19 (Levy et al., 2022). In a recent investigation by Beckman et al. (2023), JAK-STAT inhibition by fedratinib and ruxolitinib provided prevention of increased leukocyte-endothelial adherence and reduced upregulation of prothrombotic factors.

## 5 Conclusion and future perspectives

Cytokine storm, a significant feature, and influencer in the patient of COVID-19 attracted several researchers to focus on the multi-targeting JAK-STAT pathway signaling. Based on the history of utilization of Jakinibs in various autoimmune and inflammatory disorders with similar cytokine release patterns, Jakinibs were repurposed and investigated in several clinical trials. Jakinibs demonstrated promising potential when utilized as a drug repurposing strategy for the treatment of COVID-19 associated cytokine release syndrome. Baricitinib has emerged as a victorious candidate in the treatment of COVID-19, given the availability of convincing data. However, the precise timing of the treatment is still unknown, and future studies are needed. Concerning Jakinibs utilization in COVID-19 management, documented evidence suggests the non-involvement of JAK2 inhibitors in interfering cell signaling by type-I interferons required for the antiviral immunity of the host cells. Selective JAK2 inhibitors represent an attractive therapeutic option for preventing COVID-19-associated cytokine storm with minimal effects on the host immune system-mediated antiviral and antibacterial activities. Several JAK2 inhibitors, including pacritinib, lestaurtinib, ilginatinib, and gandotinib, are either approved or under investigation in clinical trials for various immunological disorders; they may also be investigated for treating COVID-19.

During the COVID pandemic, several drug repurposing strategies were investigated, including anti-viral, anti-protozoal, anti-inflammatory, and fibrinolytic agents. Concurrently, different drug combinations were also tried to treat COVID-19. Given the advantage of combination therapy over monotherapy concerning reducing the risk of adverse events and drug resistance, various groups started evaluating Jakinibs with antivirals, corticosteroids, and other immunomodulatory agents. Based on preliminary results, the combination approach provides better clinical outcomes in hospitalized COVID-19 patients. Jakinibs with remdesivir and corticosteroids have demonstrated positive results. More results are required to establish the safety and efficacy of suitable combination modalities with jakinibs in the clinical setting. Though Jakinibs alone are highly efficacious, their high cost and risk of serious adverse events limit their application. Combining low-cost alternative medication that affects the JAK-STAT pathway in COVID-19 patients may improve treatment outcomes and decrease adverse effects.

In the last 3 decades, Jakinibs has grown drastically as a pharmaceutical class to cover a large spectrum of difficult-to-manage illnesses and diseases. Surprisingly, Jakinibs exhibit safety comparable to multiple biologics, sometimes more efficacious. However, despite the several advantages of Jakinibs over biologics, such as multi-target blocking and ease of administration, there are serious concerns related to the management and treatment of serious adverse effects, including cardiovascular disorders, thrombotic complications, malignant tumors and risk of deaths, which are more significant than the use of biologics.

Regarding safety concerns associated with Jakinibs monotherapy, there is no solid evidence associating Jakinibs with a higher risk of adverse events of any grade and secondary infections in COVID-19 patients. The possible reason for the lower incidence rate of adverse events is the short duration of treatment of COVID-19. Various metanalyses have suggested clinical improvement in the form of lower mortality rate, higher recovery rate, reduced disease progression, and a lower rate of mechanical ventilation use in hospitalized COVID-19 patients. These results were based on relatively small sample size, and head-to-head clinical trial comparisons are lacking. Therefore, further data must be accumulated to establish risk-free utilization of Jakinibs. Thus, there prescription and administration of Jakinibs need to be handled by specialists who can identify the potential patient cohorts who might benefit from Jakinibs. Also, proper screening and monitoring are essential before and during the treatment to judge the progress of therapeutic benefits.

The last year, 2022, has witnessed remarkable success in recognizing and validating Jakinibs application in multiple autoimmune and inflammatory disorders. Up to 2021, the FDA and EMA authorized Jakinibs such as ruxolitinib, tofacitinib, baricitinib, upadacitinib, peficitinib, fedratinib, delgocitinib, filgotinib, and oclacitinib for the treatment of various therapeutic conditions. These conditions included myelofibrosis, ulcerative colitis, rheumatoid arthritis, acute and chronic GVHD, atopic dermatitis (human and canine), psoriatic arthritis, juvenile idiopathic arthritis, and ankylosing spondylitis. In 2022, three new Jakinibs such as abrocitinib (atopic dermatitis), pacritinib (myelofibrosis), and deucravacitinib (plaque psoriasis), got approval from FDA for the first time, expanding the Jakinibs portfolio to a total of 10 Jakinibs approved for human and veterinary use, in United states. A total of twelve Jakinibs have been approved by different regulatory authorities (FDA, EMA, MHLW, TGA, etc.) worldwide for various autoimmune and inflammatory conditions. Similarly, in 2022, five new indications have been added to the therapeutic basket of Jakinibs, namely, non-segmental vitiligo (ruxolitinib), atopic dermatitis (baricitinib), plaque psoriasis (deucravacitinib), non-radiographic axial spondyloarthritis (upadacitinib) and COVID-19 (baricitinib). In the last 30 years, jakinib drug development has undergone drastic translation from bench to clinic. These agents have become an integral part of treatment for a heterogenous group of ailments either as an alternative treatment for the conditions in which other biologics failed or as the sole mainstay treatment of conditions without prior approved therapeutic agents. We hope the current year of Jakinibs will be as exciting as last year’s, and more Jakinibs will come along to address the unmet needs of many more autoimmune-inflammatory conditions.

## Glossary

PCR: Polymerase chain reaction
COVID-19: coronavirus disease
SARS-CoV-2: severe acute respiratory syndrome coronavirus 2
Acyl CoA: Acyl coenzyme A
ATP: adenosine triphosphate
GM-CSF: Granulocyte-macrophage colony-stimulating factor
IC50: Half maximal inhibitory concentration
BID: two times a day
QD: once a day
IL: interleukin
IFN: interferons
MCP-1: Monocyte chemoattractant protein-1
B-cell: B lymphocytes
T-cell: T lymphocytes
NK-Cell: Natural killer cells
EGFR: epidermal growth factor receptor
VEGF: Vascular endothelial growth factor
FGFR: fibroblast growth factor receptors
PDGFR: platelet-derived growth factor receptors
PDGF: platelet-derived growth factor
VEGFR: vascular endothelial growth factor receptors
VEGF: vascular endothelial growth factor
AAK1: AP2-associated protein kinase 1
GAK: Cyclin G-associated kinase
IRAK1: Interleukin-1 receptor-associated kinase 1
TLR8: Toll-like receptor 8
SaO2: arterial oxygen saturation
ICU: intensive care unit
EULAR: European Alliance of Associations for Rheumatology
q24 h: every 24 h
Pgp: P-glycoprotein 1
CAR-T: Chimeric antigen receptor-T cell
BRD4: Bromodomain-containing protein 4
MTD: maximum tolerated dose
DMARDs: Disease-modifying antirheumatic drugs
